# Primary Ewing sarcoma/peripheral primitive neuroectodermal tumors in the cranial bone and mobile spine: what is the difference?

**DOI:** 10.1186/s12893-021-01452-4

**Published:** 2022-01-08

**Authors:** Jun Chen, Shi-Zhou Wu, Jie Tan, Qing-Yi Zhang, Bo-Quan Qin, Yu Wang, Hui Zhang

**Affiliations:** 1 Department of Orthopaedic Surgery, West China Hospital, Sichuan University, Chengdu, Sichuan 610041 People’s Republic of China; 2 Department of Neurosurgery, Tongji Hospital, Tongji Medical School, Huazhong University of Science and Technology, Wuhan, Hubei 430030 People’s Republic of China

**Keywords:** Ewing sarcoma, Primitive neuroectodermal tumors, Cranial bone, Mobile spine, Prognosis

## Abstract

**Background:**

Primary Ewing sarcoma (ES)/peripheral primitive neuroectodermal tumors (pPNETs) are aggressive bone tumors that rarely occur in the axial skeleton, including the cranial bone and mobile spine. The purpose of this study was to investigate whether there were any differences in patient characteristics, treatment strategies, and outcomes between patients with ES/pPNETs of the cranial bone and those with ES/pPNETs of the mobile spine.

**Methods:**

A retrospective study was performed on 33 patients with ES/pPNETs who had been surgically treated and pathologically confirmed at our institution between 2010 and 2020. Patient characteristics were compared using Fisher exact tests or independent t tests. Survival rates were estimated via Kaplan–Meier survival analysis and compared using log-rank tests.

**Results:**

Thirteen patients had ES/pPNETs of the cranial bone (39.4%), while 20 patients had ES/pPNETs of the mobile spine (60.6%). Patients with ES/pPNETs of the cranial bone had a younger mean age (14.8 vs 22.6 years; *p* = 0.047) and longer mean disease duration (2.5 vs 1.9 months; *p* = 0.008) compared with those of patients with ES/pPNETs of the mobile spine. Kaplan–Meier analysis showed that gross total resection (GTR) and radiotherapy resulted in a longer median survival time. The overall survival rates and progression-free survival rates of patients with ES/pPNETs of the cranial bone versus those of the mobile spine were not significantly different (*p* = 0.386 and *p* = 0.368, respectively).

**Conclusions:**

Patients with ES/pPNETs of the cranial bone were younger compared to patients with ES/pPNETs of the mobile spine. There was no significant difference in the prognosis of patients with ES/pPNETs of the cranial bone versus those of the mobile spine. Taken together, our findings suggest that GTR and radiotherapy offer the best prognosis for improved long-term survival.

## Background

Ewing sarcoma (ES)/peripheral primitive neuroectodermal tumors (pPNETs) are the second most common primary sarcoma of bone [1]. ES/pPNETs occur primarily in children and adolescents, and they exhibit an aggressive nature with a high tendency to form rapid growths and distant metastases [2, 3]. Most primary ES/pPNETs occur in the pelvis and extremities [4]. Tumors located in the cranial bone and mobile spine account for approximately 1–4% and 8% of all primary sites, respectively [5, 6]. Involvement of the cranial bone or mobile spine usually indicates progressive or metastatic disease [5, 7].

Although most modern treatments—including combination surgery, postoperative radiotherapy, and chemotherapy—have improved the prognosis of primary ES/pPNETs over the past decades [8], the survival rate of patients with ES/pPNETs and unresectable disease remains poor, especially in those with large tumors occurring in the cranial bone or mobile spine [5, 9]. Previous studies have shown that the 5-year overall survival for patients with primary ES/pPNETs is 65–75% [10]. However, for patients with axial locations, the outcome is very poor [11, 12]. In addition, some studies suggest that axial primary ES/pPNETs have a more aggressive phenotype due to the microenvironmental milieu of the axial skeleton [12, 13].

Primary ES/pPNETs are poorly differentiated malignant bone tumors that rarely occur in the axial skeleton, including the cranial bone and mobile spine. These sites are often grouped together due to their rarities as primary ES/pPNET localizations. Limited information on primary ES/pPNETs of the cranial bone and mobile spine has been published due to the rarity of these diseases, and most related information about the outcome of unusual sites of ES/pPNETs derives from case reports or case series, in which no comparisons have been reported on any differences between these localizations. Hence, the purpose of the present study was to investigate whether there was any difference in patient characteristics, treatment strategies, and outcomes between patients with ES/pPNETs of the cranial bone and those with ES/pPNETs of the mobile spine.

## Materials and methods

A retrospective study was performed on 33 patients with ES/pPNETs who had been surgically treated and pathologically confirmed at our institution between 2010 and 2020. Patients with tumor dissemination from intracranial locations or any evidence of metastatic disease were excluded. The pathological diagnosis of primary ES/pPNETs was confirmed by three experienced independent pathologists. The following clinical characteristics of the 33 patients were collected: disease duration, resection mode, tumor pathology, adjuvant therapy, postoperative tumor recurrence, distant metastasis, and survival. We also measured progression-free survival (PFS) and overall survival (OS). PFS was defined as the period from the date of first operation to the date of tumor recurrence, distant metastasis, and/or regrowth. OS was defined as the period from the first operation to the date of death from any cause.

For the cranial-bone group, a large skin flap was raised. Dissection was performed until normal bone on all sides could be exposed. The affected bone was removed as much as possible. The dura mater was not opened if it was not invaded by the tumor. However, if it was invaded, the infiltrated dura mater was completely removed and repaired by using temporalis fascia, fascia lata, or pericranium. For the mobile-spine group, surgical treatment was performed by posterior approach, anterior approach, and combined approach. Single-level of the affected spine in 6 cases, 2-level in 12 cases, and 3-level in 2 cases. Posterior reconstruction was performed using rods, pedicle screws, and transverse connectors. The posterior spinal instrumentation was adjusted to slightly compress the inserted cage. Anterior reconstruction was conducted using a titanium mesh cage filled up with morcelized autograft harvested from the rib or iliac crest. When the lesion was located in the lower lumbar spine, invasion of the adjacent tissue was present but not safely reachable posteriorly, a combined anterior–posterior approach was performed.

Adjuvant systemic treatment consisting of radiotherapy and/or chemotherapy was performed depended on the patient’s tolerance, preference, age, and postoperative Karnofsky performance status (KPS) scores. After initial surgery, adjuvant chemotherapy was advised to patients with postoperative KPS scores ≥ 70. Adjuvant radiotherapy was performed two weeks after the initial operation in patients more than 3 years old whose guardians were not willing for them to receive postoperative chemotherapy. In patients underwent postoperative chemotherapy, radiotherapy was performed depended on the patient’s tolerance, age, and parental preference.

Gene/locus/chromosomal gains or losses were cytogenetically investigated by applying FISH probes (No. 03 N59–020, Abbott Molecular Inc., Wuhan Zhongji Scientific Instruments Co. Ltd. Wuhan, China.) to histologically confirmed paraffinembedded tumor specimens. In addition, the dual color Break Apart FISH probe was applied to detect gene-related rearrangements at the EWSR1 locus. The probe consisted of a mixture of two FISH DNA probes. Specific experimental procedures follow the manufacturer's protocol.

Follow-up data for all patients with primary ES/pPNETs were primarily obtained through office visits, supplemented by telephonic interviews. The length of follow-up was recorded as the period from the date of first operation to death, or until February 2021 for patients who have survived. The mean follow-up period was 31.8 months (range, 3–117 months). The follow-up ended on February 26, 2021.

### Statistical analysis

IBM SPSS Statistics version 20.0 (IBM Corp., Armonk, New York, USA) was applied for all statistical analyses. Patient characteristics were compared using Fisher exact tests or independent t tests. Survival rates were estimated via Kaplan–Meier survival analysis and compared using log-rank tests. Kaplan–Meier analysis was also used to calculate the median survival time (MST), and the MST in different treatment groups was compared using log-rank tests. Statistical significance was defined as a *P* value < 0.05.

## Results

**Fig. 1:**
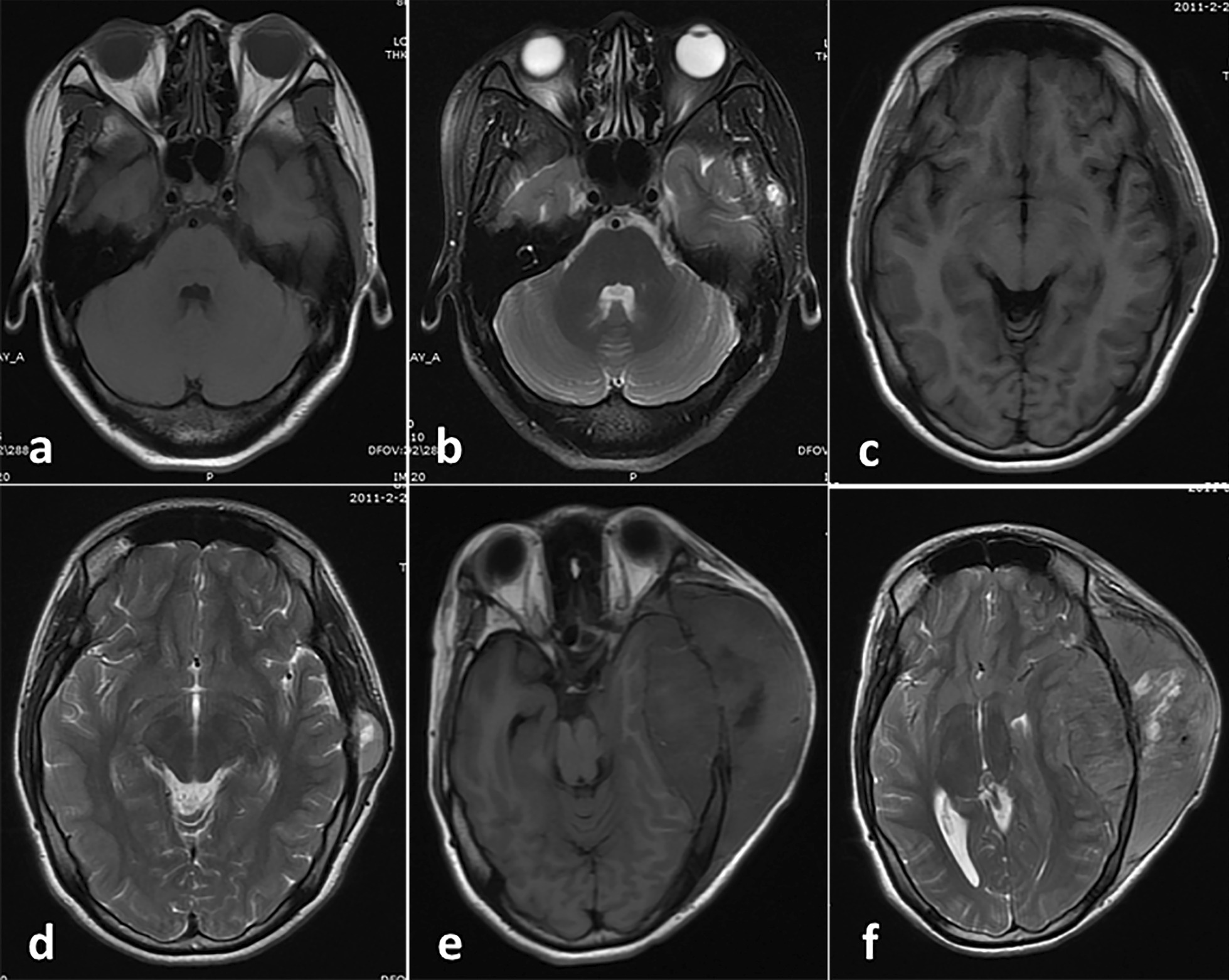
A case of lesion located in the cranial bone with bony destruction. **a**, **b** Four months before surgery, a solid appearance was observed and the border was relatively clear. **c**, **d** One week before surgery, the tumor is larger than it was three months ago. **e**, **f** Five months after initial surgery, tumor recurrence was observed

As shown in Table 1, 13 of the primary tumors arose in the cranial bone (39.4%), whereas 20 tumors arose in the mobile spine (60.6%). Bony destruction or rarefaction at certain locations was noted in all patients (Fig. 1). The 13 cases of primary ES/pPNETs of the cranial bone occurred in six females and seven males. The 20 cases of primary ES/pPNETs of the mobile spine occurred in eight females and 12 males. The clinical characteristics of patients in these two groups are outlined in Table 1. Patients with ES/pPNETs of the cranial bone had a younger mean age (14.8 years, range: 1–42 years) compared with patients with ES/pPNETs of the mobile spine (22.6 years, range: 2–45 years; *p* = 0.047). The clinical progression was rapid, and the mean time interval from symptom onset to hospital admission for patients with ES/pPNETs of the mobile spine (1.9 months, range: 0.2–6.0 months) was shorter than that for patients with ES/pPNETs of the cranial bone (2.5 months, range: 0.2–7.0 months; *p* = 0.008). For the cranial-bone group, the mean tumor maximum diameter at diagnosis was 5.1 cm (range, 3.0–9.0 cm), while that for the mobile spine group was 6.2 cm (range, 4.0–10.0 cm). The predominant presenting symptom for both groups was localized pain, as noted in nine patients (69.2%) with ES/pPNETs of the cranial bone and in 12 patients (60.0%) with ES/pPNETs of the mobile spine. Five patients with ES/pPNETs of the mobile spine presented with neurologic symptoms leading to emergency decompressive operation. Neurological deficits were the presenting symptoms in seven patients (35.0%) within the mobile spine group, consisting of extremity paresthesias or weakness.

**Table 1:** Summary of clinical characteristic and course classified by primary site

Variables	Cranial bone, n = 13	Mobile spine, n = 20	*P*-value
Age			
Mean (range), years	14.8 (1–42)	22.6 (2–45)	0.047
Gender			
Male	7 (53.8%)	12 (60.0%)	0.727
Female	6 (46.2%)	8 (40.0%)	
Maximum tumor diameter: mean (range), (cm)	5 (3.0–9.0)	6 (4.0–10.0)	0.712
Disease duration: mean (range), months	2.5 (0.2–7.0)	1.9 (0.2–6.0)	0.008
Predominant presenting symptom			
Pain	9 (69.2%)	12 (60.0%)	
Neurological deficit	2 (15.4%)	7 (35.0%)	
Mode of installation of symptoms			
Acute or subacute	9 (69.2%)	13 (65.0%)	
Progressive	4 (30.8%)	7 (35.0%)	
Number of recurrences	8 (61.5%)	11 (55.0%)	0.710
Distant metastasis	2 (15.4%)	7 (45.0%)	0.263
Median survival time: months	19	25	0.386
2-Year progression-free survival rate (%)	23.1	35.0	0.368
1-Year survival rate (%)	76.9	80.0	0.581
2-Year survival rate (%)	46.2	60.0	0.284
5-Year survival rate (%)	20.5	29.2	0.558
KI-67 index: mean (range)	41.0 (2–70)	33.9 (3–80)	0.614

**Fig. 2:**
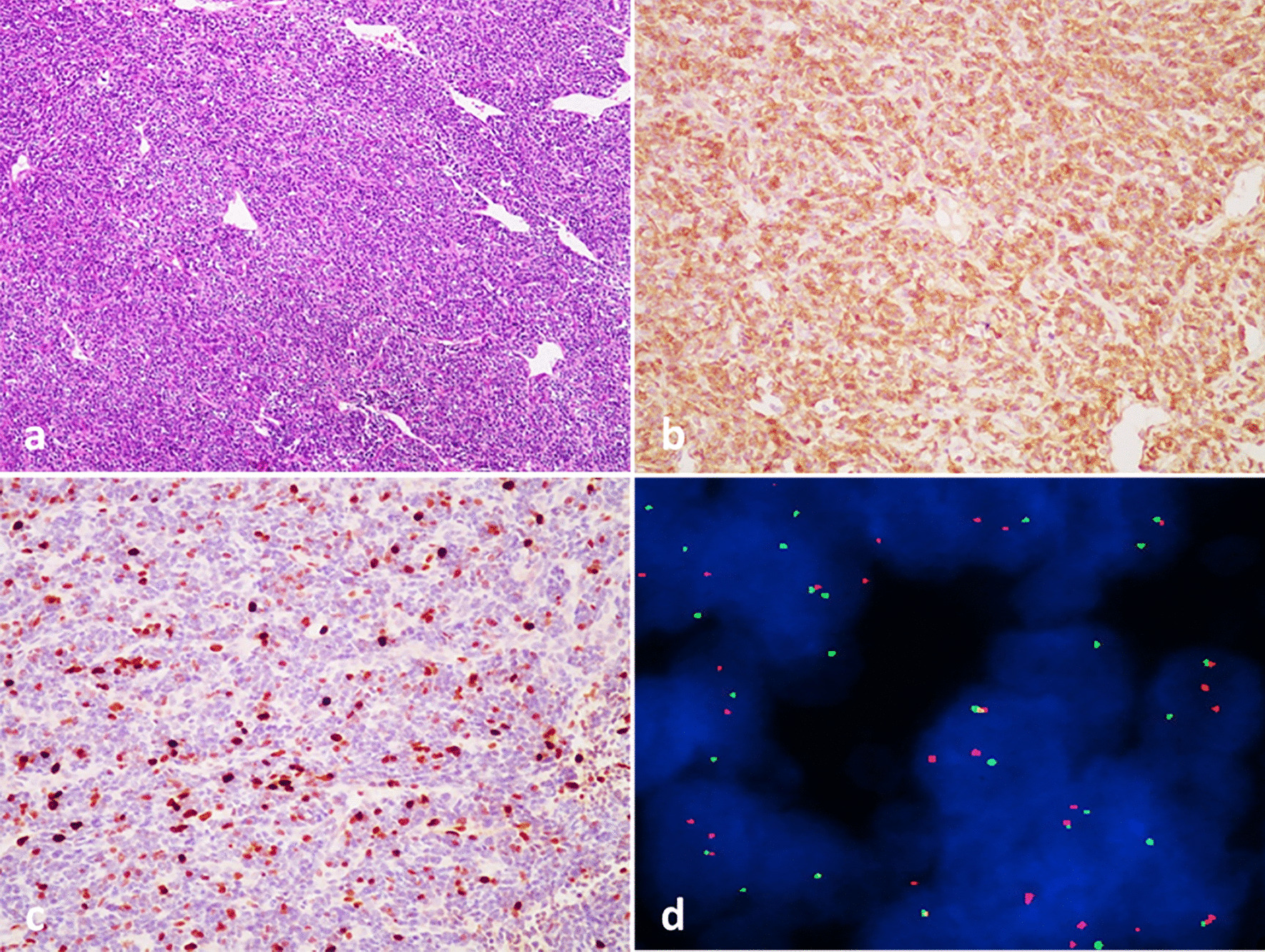
Histopathological, immunohistochemical, and cytogenetic examination of bony ES/pPNET. **a** Small, round-to-oval tumor cells with prominent perivascular arrangement (hematoxylin–eosin-stained, × 100). **b** Immunohistochemical staining showed strong positivity for CD99 (× 100). **c** Immunohistochemistry utilizing anti-MIB-1 (Ki-67) antibodies revealed a high proliferative index (× 100). **d** Representative image of FISH labeling using EWS break-apart probes

Light microscopic histological examination of specimens from all cases revealed malignant round-cell tumors involving bone (Fig. 2). Immunohistochemical studies showed that all patients were positive for CD99 (Fig. 2). Friend leukemia virus integration 1 (FLI-1) was positive in 65% of the patients. Immunohistochemistry using anti-MIB-1 (Ki-67) antibodies revealed a high proliferative index in most patients (Fig. 2). Fluorescent in situ hybridization (FISH) was performed in six cases, and EWS/FLI-1 fusion was found in four cases (Fig. 2). However, a corresponding FISH and chromosomal analysis were not performed in the other 27 cases.

The treatment modalities and clinical outcomes of the patients in each group are presented in Tables 2 and 3. In addition, the clinical outcomes in all combined cases are presented in Table 4. Gross total resection (GTR) was achieved in seven patients (53.8%) with ES/pPNETs of the cranial bone and in 12 patients (60.0%) with ES/pPNETs of the mobile spine. Subtotal resection was achieved in six patients (46.2%) with ES/pPNETs of the cranial bone and in eight patients (40.0%) with ES/pPNETs of the mobile spine. Tumor recurrence was noted in eight patients (61.5%) with ES/pPNETs of the cranial bone and in 11 patients (55.0%) with ES/pPNETs of the mobile spine. Distant metastasis occurred in seven patients (35.0%) with ES/pPNETs of the mobile spine. The distant metastatic sites were in the lungs in four cases, in the sternum in two cases, in the rib in one case, and in the mediastinum in one case. For the cranial-bone group, only two cases had distant metastasis. However, local recurrence or metastatic status did not differ by group (*p* = 0.710 and *p* = 0.263, respectively).

**Table 2:** Summary of clinical outcomes of primary Ewing sarcoma/peripheral primitive neuroectodermal tumors in the cranial bone

Parameters	Number	Progression-free survival		Overall survival	
		Median time (month)	*p* value	Median time (month)	*p* value
Resection mode					
GTR/ without GTR	7/6	15 vs. 5	0.019	30 vs. 13	0.021
Ki-67 index					
≤ 30 / > 30%	5/8	8 vs. 7	0.972	19 vs. 18	0.916
Postoperative radiotherapy					
Yes / no	7/6	15 vs. 5	0.008	29 vs. 6	0.008
Postoperative chemotherapy					
Yes / no	7/6	8 vs. 7	0.584	25 vs. 18	0.948
GTR + radiotherapy					
Yes / no	5/8	29 vs. 6	0.004	30 vs. 13	0.006
GTR + chemotherapy					
Yes/no	4/9	15 vs. 7	0.053	19 vs. 14	0.145

*GTR* gross total resection

**Table 3:** Summary of clinical outcomes of primary Ewing sarcoma/peripheral primitive neuroectodermal tumors in the mobile spine

Parameters	Number	Progression-free survival		Overall	survival
		Median time (month)	*p* value	Median time (month)	*p* value
Resection mode					
GTR/ without GTR	12/8	15 vs. 5	0.020	37 vs. 13	0.004
Ki-67 index					
≤ 30 / > 30%	9/11	15 vs. 13	0.256	36 vs. 24	0.131
Postoperative radiotherapy					
Yes / no	12/8	25 vs. 9	0.005	36 vs. 13	0.002
Postoperative chemotherapy					
Yes / no	11/9	17 vs. 13	0.528	32 vs. 25	0.652
GTR + radiotherapy					
Yes / no	10/10	45 vs. 8	0.003	61 vs. 13	< 0.001
GTR + chemotherapy					
Yes/no	7/13	15 vs. 14	0.272	37 vs. 25	0.159

*GTR* gross total resection

**Table 4:** Summary of clinical outcomes of primary Ewing sarcoma/peripheral primitive neuroectodermal tumors in the cranial bone and mobile spine

Parameters	Number	Progression-free survival		Overall survival	
		Median time (month)	*p* value	Median time (month)	*p* value
Resection mode					
GTR/ without GTR	19/14	15 vs. 5	0.001	37 vs. 13	< 0.001
Postoperative radiotherapy					
Yes / no	19/14	25 vs. 7	< 0.001	37 vs. 13	< 0.001
Postoperative chemotherapy					
Yes / no	18/15	15 vs. 10	0.291	25 vs. 24	0.603
GTR + radiotherapy					
Yes / no	15/18	45 vs. 7	< 0.001	61 vs. 13	< 0.001
GTR + chemotherapy					
Yes/no	11/22	15 vs. 10	0.053	37 vs. 24	0.062

*GTR* gross total resection

**Fig. 3:**
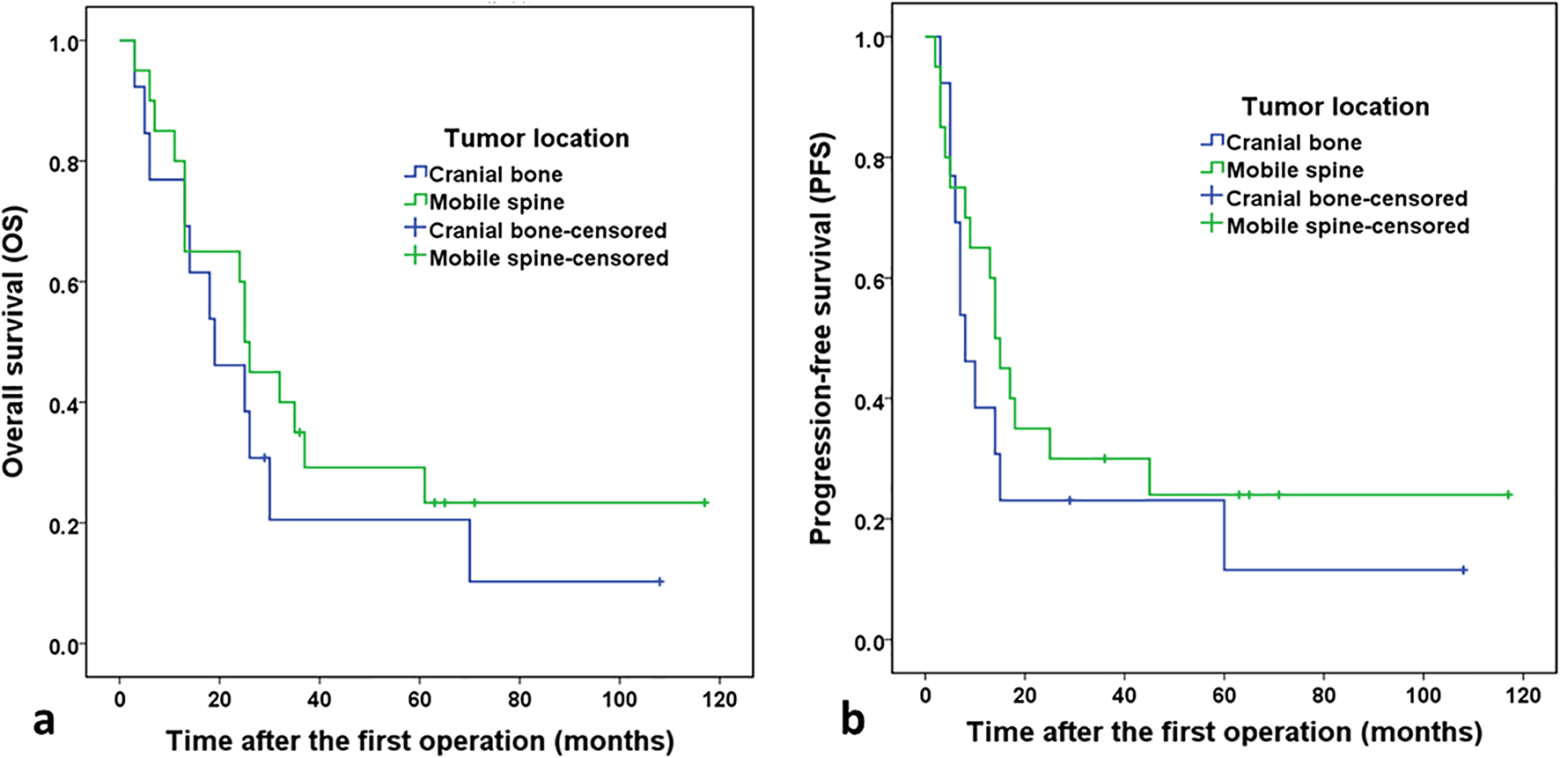
Overall survival and progression-free survival based on tumor location. Overall survival (**a**) tends to be lower in patients with ES/ pPNETs of the cranial bone than in those with ES/ pPNETs of the mobile spine, although the difference is not statistically significant. Progression-free survival (**b**) tends to be lower in patients with ES/ pPNETs of the cranial bone than in those with ES/ pPNETs of the mobile spine, although the difference is not statistically significant

**Fig. 4:**
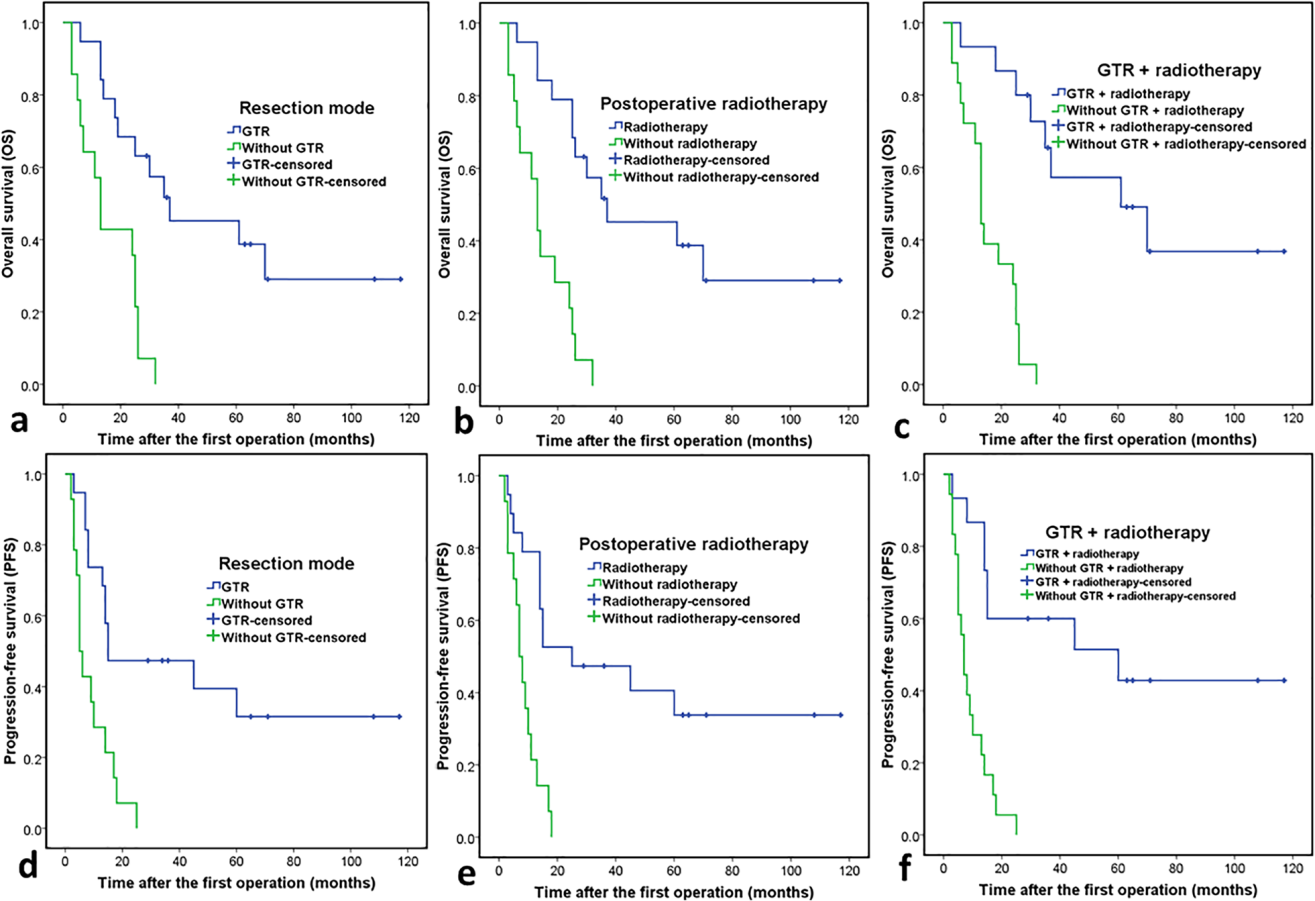
Kaplan Meier curves of overall survival and progression-free survival. Kaplan Meier curves of overall survival (**a**) for the resection mode. Kaplan Meier curves of overall survival (**b**) of patients treated with and without radiotherapy. Kaplan Meier curves of overall survival (**c**) for patients with and without GTR + radiotherapy. Kaplan Meier curves of progression-free survival (**d**) for the resection mode. Kaplan Meier curves of progression-free survival (**e**) for patients treated with and without radiotherapy. Kaplan Meier curves of progression-free survival (**f**) for patients treated with and without GTR + radiotherapy

Kaplan–Meier survival analyses for OS and PFS of patients from each group are shown in Fig. 3. In addition, Kaplan–Meier survival analyses for OS and PFS of patients from all combined cases are shown in Fig. 4. The OS rates of patients with ES/pPNETs of the cranial bone at 1, 2, and 5 years were 76.9%, 46.2%, and 20.5%, respectively. The PFS rates of patients with ES/pPNETs of the cranial bone at 1, 2, and 5 years were 38.5%, 23.1%, and 11.5%, respectively. The OS rates of patients with ES/pPNETs of the mobile spine at 1, 2, and 5 years were 80.0%, 60.0%, and 29.2%, respectively. The PFS rates of patients with ES/pPNETs of the mobile spine at 1, 2, and 5 years were 60.0%, 35.0%, and 24.0%, respectively. However, there were no significant differences in OS or PFS rates between the two groups (*p* = 0.386 and *p* = 0.368, respectively).

The results of Kaplan–Meier analysis are shown in Tables 2 and 3. For the cranial-bone group, patients who received GTR had a significantly longer MST (30 months) compared with that of patients without GTR (13 months; *p* = 0.021); patients who underwent radiotherapy also had a significantly longer MST (29 months) compared to that of other patients (six months; *p* = 0.008). For the mobile-spine group, patients who received GTR had a significantly longer MST (37 months) compared to that of patients without GTR (13 months; *p* = 0.004); patients who underwent radiotherapy also had a significantly longer MST (36 months) compared to that of other patients (13 months; *p* = 0.002). Patients who received both GTR and radiotherapy, with or without chemotherapy, had the longest MST (61 months).

## Discussion

The purpose of the present study was to investigate whether there were any differences in the characteristics of tumors in patients with ES/pPNETs of the cranial bone versus those of the mobile spine. These two sites are often grouped as ES/pPNET localizations because of their rarity. Although many studies have investigated bony ES/pPNETs, few studies have differentiated between ES/pPNETs of the cranial bone versus those of the mobile spine, and no previous study has compared these two entities in a similarly treated population. Here, we sought to retrieve data of these unusual presentations and assess their characteristics, treatment strategies, and outcomes.

### Epidemiology

ES/pPNETs are very rare entities that account for approximately 10% of primary malignant bone tumors [5]. Most of these primary tumors originate from long bones (47%), the pelvis (19%) or ribs (12%) [5]. Tumors located in the cranial bone and mobile spine account for approximately 1–4% and 8% of all primary sites, respectively [5, 6, 14]. In our present study, 13 of the primary tumors arose in the cranial bone (39.4%) and 20 tumors arose in the mobile spine (60.6%). Our present findings were mostly consistent with previous findings in that primary ES/pPNETs were more likely to occur in the mobile spine than in the cranial bone [5, 6].

In our present study, male patients were more frequently affected than female patients, which is consistent with previous reports [5, 15]. Previous studies have asserted that primary ES/pPNETs are most prevalent in children and adolescents [2, 5, 16], which our present findings corroborate. Patients with ES/pPNETs of the cranial bone and mobile spine who enrolled in our present study had a mean age of 14.8 and 22.6 years, respectively. In addition, the age of patients with ES/pPNETs of the cranial bone was found to be significantly younger than that of patients with ES/pPNETs of the mobile spine (*p* = 0.047).

### Clinical presentation

The clinical presentation of primary ES/pPNETs of the cranial bone varies according to tumor sites and often involves surrounding structures. The most common neurological signs in patients with ES/pPNETs of cranial bones are headaches [5, 17], as was noted in 69.2% of the patients in our present study. Primary ES/pPNETs of the mobile spine are aggressive tumors that rapidly enlarge over a short period of time, compressing the surrounding structures and leading to corresponding symptoms [18]. In our present study, the most common preoperative clinical symptom was localized pain (60.0%), followed by extremity paresthesias or weakness (35.0%), findings that are largely consistent with those of previous reports [18, 19]. In addition, five patients with ES/pPNETs of the mobile spine presented with neurological symptoms leading to emergency decompressive operation. The clinical progression was rapid, and the mean time interval from symptom onset to hospital admission for patients with ES/pPNETs of the mobile spine was significantly shorter than that for patients with ES/pPNETs of the cranial bone (*p* = 0.008). However, there was no significant difference in the mean tumor maximum diameter at diagnosis between mobile-spine ES/pPNETs and cranial-bone ES/pPNETs (*p* = 0.712).

### Diagnosis

ES/pPNETs are poorly differentiated, small, round, blue cellular tumors with hyperchromatic nuclei [20]. Accurate diagnosis depends on immunohistochemistry and molecular genetic analysis. Previous studies showed that membranous expression of MIC2 glycoprotein (CD99) was detected in 97% of patients, and the most sensitive and specific detection method for the diagnosis of ES/pPNET was a combination of CD99 and FLI-1 immunohistochemistry [21, 22]. In our present study, immunohistochemical staining showed that the tumor cells expressed CD99 in all cases, consistent with the diagnosis of ES/pPNETs. FLI-1 was found in 65% of the patients, which further confirmed the diagnosis of ES/pPNETs. However, our study showed that there was no significant difference in the mean Ki-67 labeling index between cranial-bone ES/pPNETs and mobile-spine ES/pPNETs (*p* = 0.614).

### Treatment modalities

Unfortunately, there are currently no standard therapeutic strategies for primary ES/pPNETs of the cranial bone or mobile spine, and little is known about the benefits of different treatment modalities. In the present study, we reported a large series of primary bony ES/pPNETs in 33 patients. Treatments for bony ES/pPNETs consist of a multimodal approach, including surgery, chemotherapy and/or radiotherapy. At present, the cornerstone of therapy for bony ES/pPNETs is the radical excision of the tumor [5, 9, 23, 24]. Although local recurrence and metastases may occur even with GTR, radical resection of a mass is still recommended to prevent further neurological decline, relieve pain, achieve sufficient volume reduction for further adjuvant therapies, and effectively prolong survival [25]. In our series, management of the two groups was along similar lines, using surgery and radiotherapy in the majority of cases. In each group, the MST of patients treated with GTR was significantly longer than that of patients treated without GTR. These data suggest that GTR may improve survival in patients with ES/pPNETs of the cranial or mobile spine. Therefore, we believe every effort should be made to achieve radical excision, as long as it is safe to do so.

Because of the paucity of data in patients with bony ES/pPNETs, the benefit of adjuvant therapy remains unclear. Previous study has showed that adjuvant chemotherapy with total or subtotal tumor resection improved the five-year survival rate for localized bony ES/pPNETs [1]. A recent study found that Sphinosine-1-phosphate receptors 1 and 2 in ES/pPNETs vasculature can be modulated to normalize tumor vessels and improve chemotherapy efficacy [26]. In addition, presurgical chemotherapy can induce shrinkage of the primary tumor, which contributes to effective local and systemic control [27, 28]. Gomez-Brouchet et al. found that good chemotherapy response was associated with lower risk of local recurrence [29]. However, Wan et al. reported that postoperative chemotherapy did not decrease the recurrence rate for spinal ES/pPNETs [15]. In our series, there was no significant difference in the MST of patients treated with adjuvant chemotherapy compared to that of patients treated without adjuvant chemotherapy.

In cases in which total en-bloc resection is not possible due to the large size of the tumor and its anatomical complexity, postoperative radiotherapy is usually selected. Previous study has reported that combined surgery plus radiotherapy is acceptable for patients who unexpectedly have positive margin [30]. In recent years, international guidelines have suggested postoperative radiotherapy in case of positive surgical margins [31, 32]. Previous studies have reported that the 5-year local control rate of patients treated with postoperative radiotherapy ranged from 63 to 69.2% [33, 34]. In our series, the MST of patients treated with postoperative radiotherapy was significantly prolonged in each group. Hence, we believe that postoperative radiotherapy is an important treatment modality for patients with ES/pPNETs of the cranial bone or mobile spine, and we suggest that radiotherapy should be conducted as soon as possible after operation. Previous studies have detected higher local relapse rates after radiotherapy alone than after combined surgery and radiotherapy [35–37]. Our present findings showed that combined GTR plus radiotherapy was an effective local control modality for ES/pPNETs of the cranial bone or mobile spine. Our results showed that a combination of GTR and radiotherapy significantly improved both PFS and OS.

### Recurrence

Recurrence of bony ES/pPNETs is most common within two years of initial surgery (approximately 80%) [38]. In addition, higher rates of local failure are seen in patients older than 14 years who have maximum tumor diameters greater than 8 cm [39]. The OS of patients with recurrent ES/pPNETs is poor. A previous study has shown that the 5-year survival rate following recurrence is approximately 10–15% [5]. In our series, patients with tumors of the cranial bone (61.5%) had a slight higher recurrence rate as compared to those with ES/pPNETs of the mobile spine (55.0%), but this difference was not statistically significant (p = 0.710).

### Metastasis

Metastasis is present in approximately 25–34% of patients with ES/pPNETs (10% in bone/bone marrow, 10% in the lungs, and 5% from a combination of sites) [4, 40–42]. However, distant metastasis is rarely seen in patients with primary ES/pPNETs of the cranial bone [5, 36]. Metastatic tumors usually predict a poor prognosis, but patients with lung metastasis alone exhibit a better prognosis than patients with bone-marrow or bone metastases [42]. In our present study, there was no significant difference in the tumor metastasis rate of patients with ES/pPNETs of the cranial bone versus those of the mobile spine.

### Prognosis

A previous study has shown that patients with axial tumors have poorer outcomes than those with tumors in the rib, sternum, or clavicle [43]. In our present study, there were no significant differences in the OS or PFS of patients with ES/pPNETs of the cranial bone versus those of the mobile spine. Due to its anatomical location, patients with ES/pPNETs of the mobile spine are likely to experience symptoms when the lesion is relatively small in size and, thus, these patients often seek medical help at an earlier stage. This may explain the relatively good prognosis in spite of the difficulties associated with local treatment.

### Limitations

This is a retrospective review of a rare disease. Typical biases exist, and the statistical analysis may be limited given the small number of cases and the retrospective nature of the study. In addition, there is also concern that the correlation is an artifact of the small, uncontrolled patient group. Thus, the effective method for evaluating our conclusion is a prospective randomized trial, but this approach is complicated by the rarity of ES/pPNETs.

## Conclusions

Primary ES/pPNETs of the cranial bone and mobile spine are extremely rare, and they have an aggressive clinical course, with a high tendency for local recurrence. The ES/pPNETs of the mobile spine were more prone to distant metastases than were ES/pPNETs of the cranial bone. Patients with ES/pPNETs of the cranial bone were younger compared with patients with ES/pPNETs of the mobile bone. Our study demonstrates that the most beneficial treatment modality is GTR combined with adjuvant radiotherapy. Furthermore, our findings highlight that the prognosis of ES/pPNETs of the cranial bone and mobile spine is poor and that these two entities do not show a significant difference in terms of outcomes.

## Data Availability

The data that support the findings of this study are available from the corresponding author upon reasonable request.

## Abbreviations

ES/ pPNETs: Primary Ewing sarcoma/peripheral primitive neuroectodermal tumors
PFS: Progression-free survival
OS: Overall survival
MST: Median survival time
FISH: Fluorescence in situ hybridization
FLI-1: Friend leukemia virus integration 1
GTR: Gross total resection
